# Differential sensitivity to the acute and sensitizing behavioral effects of methylphenidate as a function of strain in adolescent and young adult rats

**DOI:** 10.1186/1744-9081-9-38

**Published:** 2013-10-17

**Authors:** Leora Yetnikoff, Andreas Arvanitogiannis

**Affiliations:** 1 Department of Pharmacological and Physiological Science, St. Louis University School of Medicine, St Louis, MO, USA; 2 Department of Psychology, Center for Studies in Behavioral Neurobiology, Concordia University, Montreal, Quebec, Canada

**Keywords:** Strain differences, Sensitization, Stimulant drugs, Sprague dawley, Spontaneously hypertensive rat, Wistar Kyoto

## Abstract

**Background:**

Behavioral effects of stimulant drugs are influenced by non-pharmacological factors, including genetic variability and age. We examined acute and sensitized locomotor effects of methylphenidate in adolescent and early adult male Sprague Dawley (SD), spontaneously hypertensive (SHR) and Wistar Kyoto (WKY) rats using a drug regimen that differentiates clearly between initial and enduring differences in drug responsiveness. We probed for strain and age differences in the sensitizing effects of methylphenidate using a cocaine challenge. Methylphenidate was administered to the rats in a non-home environment.

**Findings:**

Strain differences in sensitivity to single methylphenidate injections depend on age and change with continuing drug pretreatment. While SHR rats are more sensitive to methylphenidate relative to WKY regardless of age and pretreatment day, SHR rats become more sensitive to methylphenidate than SD rats towards the end of pretreatment during early adulthood. SD rats exhibit greater sensitivity to methylphenidate relative to the WKY group during adolescence, an effect that dissipates with continued drug pretreatment during adulthood. Remarkably, only SHR rats, regardless of age, exhibit methylphenidate-induced cross-sensitization to the behavioral effects of cocaine.

**Conclusions:**

Our findings suggest that SHR rats are more vulnerable than other strains to methylphenidate-induced cross-sensitization to cocaine, at least when methylphenidate is administered in a non-home environment. Given that SHR rats are typically used to model features of attention deficit hyperactivity disorder, these findings may have important implications for the treatment of this disorder with methylphenidate.

## Findings

Acute and chronic locomotor and neurochemical effects of stimulant drugs are determined not only by the pharmacological properties of the drug itself, but also by a multitude of non-pharmacological factors, such as genetic variability and age [1-8]. Recently, Dafny and colleagues reported strain differences in sensitization to the locomotor activating effects of methylphenidate between Sprague Dawley (SD), spontaneously hypertensive (SHR) and Wistar Kyoto (WKY) rats, the extent of which was shown to depend on age [9-11], reviewed in [12]. Because SHR rats are typically used to model features of attention deficit hyperactivity disorder (ADHD), such findings are particularly salient and warrant further exploration.

In the aforementioned studies by Dafny and colleagues [9-11], rats were pretreated with methylphenidate in their home cages and, following a short withdrawal period (2 days), were tested for sensitization with a methylphenidate challenge. Here we examined strain and age differences in the acute and cross-sensitizing behavioral effects of methylphenidate by pretreating rats with the drug in an environment other than home, as administering the drug to rats in the environment in which they live is known to attenuate the locomotor activating effects of stimulant drugs [13]. We also used a drug treatment regimen that differentiates clearly between initial and long-lasting differences in drug responsiveness. While small sensitization effects are often detected soon after drug exposure, their magnitude increases over time [14-16], indicating that neuroadaptations underlying sensitization continue to develop long after the end of repeated drug pretreatment. Sensitization effects observed at early withdrawal times are likely to rely on transient changes in receptor function [17-19], whereas enduring sensitization requires long-lasting, perhaps permanent, neuroadaptations [19,20]. Moreover, to assess the generalizability of methylphenidate-induced sensitization to illicit drugs, we probed strain and age differences in the enduring sensitizing effects of methylphenidate using a cocaine, rather than a methylphenidate challenge. This study was approved by the Concordia University Animal Research Ethics Committee.

**Figure 1:**
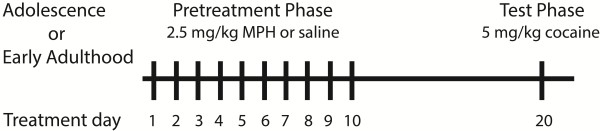
**Diagrammatic illustration of experimental procedures conducted in adolescent and early adult Spontaneously Hypertensive, Sprague Dawley, and Wistar Kyoto rats.** MPH: methylphenidate.

Figure 1 illustrates the design of the experiment. Briefly, adolescent and early adult male SD (adolescent: 100-120 g; early adult: 220-270 g), SHR (adolescent: 110-140 g; early adult: 160-180 g), and WKY (adolescent: 70-110 g; early adult: 170-190 g) rats (Charles River, St Constant, Quebec) were housed in pairs in a room with a 12-hr light-12-hr dark cycle (n = 6 for all groups unless stated otherwise). Rats arrived to the animal colony at five (adolescent) and eight (early adult) weeks of age and were given a few days to acclimate before commencement of drug pretreatment. Locomotor activity was assessed in testing chambers (40.2 × 20.2 × 30.5 cm) with transparent Plexiglas fronts, stainless steel grid floors, and wooden side walls, backs, and ceilings. A single count of locomotor activity was defined as a consecutive interruption of two photocells that were located along the longitudinal axis of each chamber, 4.8 cm above the floor. The first part of the experiment (pretreatment) lasted ten consecutive days, wherein we transported animals to the testing chambers and administered injections of saline (1 ml/kg i.p.) or methylphenidate (2.5 mg/kg i.p.; methylphenidate hydrochloride, Medisca, QC, Canada). This dose was selected because it was previously shown to be the lowest dose to elicit behavioral activation in the three rat strains used in the present study [11]. Locomotor activity was recorded for 30 min and the animals were then returned to their home cage. The second part of the experiment (test) took place ten days following the last pretreatment injection. During this test, we gave all animals a challenge injection of cocaine (5 mg/kg i.p., cocaine hydrochloride, Medisca). This low dose was chosen so as to elicit locomotion during the test, rather than stereotypy [21]. Locomotor activity was recorded for 1 hr. Two- and three-way ANOVAs were used as required to analyze behavioral effects. Significant main effects and interactions were followed by Tukey post-hoc tests.

**Figure 2:**
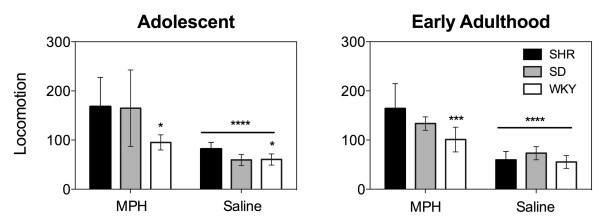
**Behavioral responses to the first injection of methylphenidate in adolescent and early adult Spontaneously Hypertensive, Sprague Dawley, and Wistar Kyoto rats.** * *p* < .05, significantly different from Spontaneously Hypertensive rat. *** *p* < .001, significantly different from Spontaneously Hypertensive rat. **** *p* < .0001, significantly different from methylphenidate-treated animals. Adult SHR group pretreated with methylphendiate, n = 5. All other groups, n = 6. SHR: Spontaneously Hypertensive rat; SD: Sprague Dawley; WKY: Wistar Kyoto; MPH: methylphenidate.

As shown in Figure 2, the first injection of methylphenidate administered to adolescent SD, SHR and WKY rats induced an increase in locomotor activity (Two-way *ANOVA* (strain × drug treatment); significant main effect of drug treatment: *F*_(1, 30)_ = 29.93, *p* < .0001). However, WKY rats, regardless of drug pretreatment, engaged in significantly less locomotor activity than SHR rats (significant main effect of strain: *F*_(2, 30)_ = 4.12, *p* = .02; significant difference between WKY and SHR rats, *p* < .05). An acute injection of methylphenidate also increased locomotor activity within early adult rats, however, the WKY group exhibited lower methylphenidate-induced locomotor activity in comparison to the SHR group (Two-way *ANOVA* (strain × drug treatment); significant main effect of drug treatment: *F*_(1, 29)_ = 71.32, *p* < .0001; significant strain × drug treatment interaction: *F*_(2, 29)_ = 4.45, *p* = .02; significant difference between methylphenidate treated SHR and methylphenidate treated WKY, *p* < .001). These results are in stark contrast to the findings of Dafny and colleagues [9-11], showing that SHR rats respond less to acute methylphenidate in comparison to SD and WKY groups. This key difference between our findings likely reflects the influence of environmental context in the modulation of the locomotor-activating effects of stimulant drugs [13] and suggests that SHR rats may be more sensitive than other strains to the attenuating effects of the home environment on stimulant-induced locomotor activity. Of note, the baseline hyperactivity typically associated with SHR rats was observed only in comparison to WKY rats, and only within the adolescent group.

**Figure 3:**
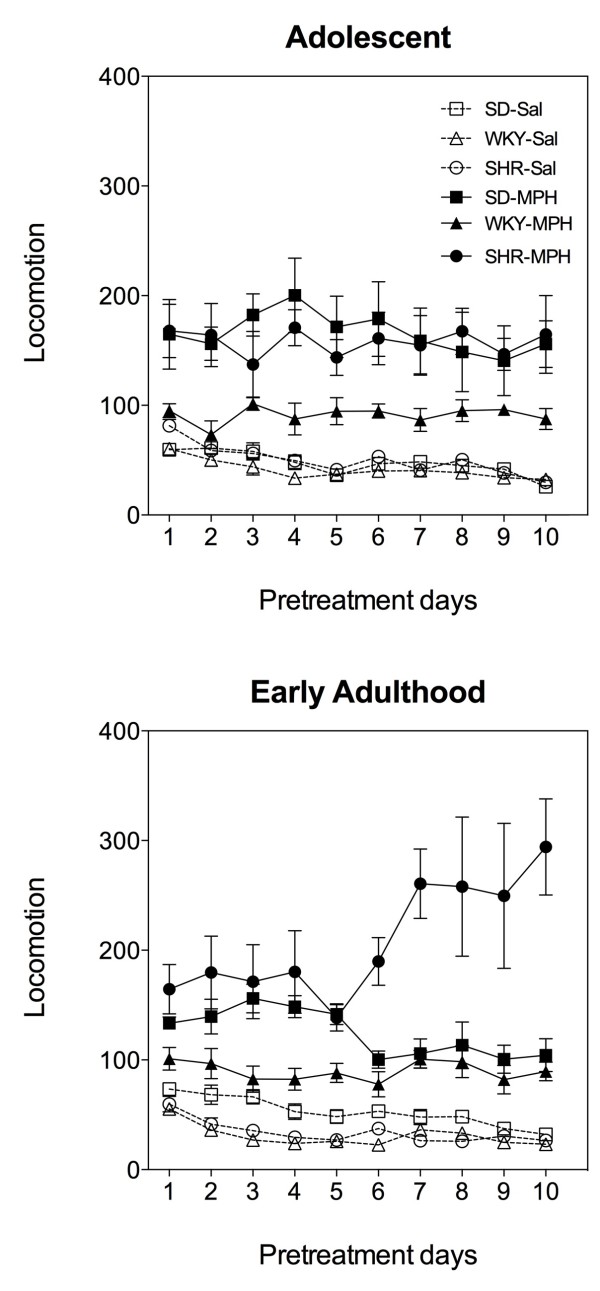
**Behavioral responses to injections of methylphenidate or saline in adolescent and early adult Spontaneously Hypertensive, Sprague Dawley, and Wistar Kyoto rats across the 10 pretreatment days.** Filled symbols represent methylphenidate-treated animals. Open symbols represent saline-treated animals. Circles, squares and triangles represent Spontaneously Hypertensive, Sprague Dawley, and Wistar Kyoto rats, respectively. Adult SHR group pretreated with methylphendiate, n = 5. All other groups, n = 6. Please see text for statistical results. SHR: Spontaneously Hypertensive rat; SD: Sprague Dawley; WKY: Wistar Kyoto; MPH: methylphenidate; Sal: saline.

Across multiple pretreatment days (Figure 3), locomotor activity remained elevated in response to methylphenidate administration as compared to saline in both adolescent and early adult SHR, WKY, and SD rats (adolescent group; Three-way *ANOVA* (strain × drug pretreatment × day): significant main effect of drug pretreatment: *F*_(1, 30)_ = 84.44, *p* < .0001; early adult group; Three-way *ANOVA* (strain × drug pretreatment × day): significant main effect of drug pretreatment: *F*_(1, 29)_ = 116.68, *p* < .0001). Within the adolescent group, the magnitude of locomotor activity elicited by methylphenidate was greater in SHR and SD rats compared to WKY rats (significant drug treatment × strain interaction: *F*_(2, 30)_ = 4.49, *p* = .02; significant differences between SHR and SD rats with WKY rats, *p* < .01). Within the early adult group, however, the picture is more complex because strain differences in methylphenidate-induced locomotor activity differ as a function of pretreatment day (significant drug pretreatment × strain × day interaction: *F*_(18, 29)_ = 4.57, *p* < .0001. Specifically, while it is clear that SHR and SD groups exhibit similar drug-induced locomotor activity on the first five pretreatment days, the SHR group (n = 5) shows a dramatically greater response to methylphenidate on the remaining days than the SD group (*p* < .01). In comparison to WKY rats, the SHR group shows significantly greater activity across all pretreatment days, excepting day 5 (day 1, *p* < .05; days 2 – 4, *p* < .01; days 6 – 9, *p* < .01; day 10, *p* < .05). SD rats exhibit similar levels of methylphenidate-induced locomotor activity to WKY rats except for pretreatment days 4 and 5, wherein SD rats exhibit greater behavioral effects than WKY rats (*p* < .05). Thus, these findings reveal that the strain differences in sensitivity to methylphenidate change with continuing drug pretreatment. While SHR rats remain more sensitive to methylphenidate relative to WKY regardless of age and daily drug exposure, SHR rats become more sensitive to methylphenidate than SD rats towards the end of pretreatment during early adulthood. In a similar way, SD rats exhibit greater sensitivity to methylphenidate relative to the WKY group during adolescence, an effect that dissipates with continued drug pretreatment during early adulthood.

**Figure 4:**
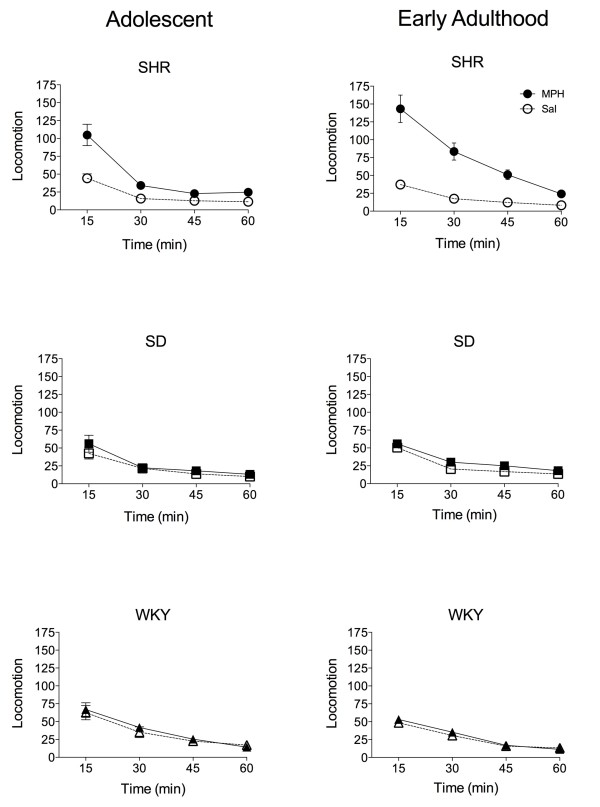
**Behavioral responses to a challenge injection of cocaine in Spontaneously Hypertensive, Sprague Dawley, and Wistar Kyoto rats pretreated with methylphenidate or saline during adolescence or early adulthood.** Only Spontaneously Hypertensive rats exhibit methylphenidate-induced behavioral cross**-**sensitization to cocaine. Adult SHR group pretreated with methylphendiate, n = 5. All other groups, n = 6. Please see text for statistical results. SHR: Spontaneously Hypertensive rat; SD: Sprague Dawley; WKY: Wistar Kyoto; MPH: methylphenidate; Sal: saline.

Results of the sensitization test are shown in Figure 4. Sensitization is defined as a greater response to the drug challenge injection in drug- versus saline-pretreated animals within each strain. It is remarkable that, at both ages, only SHR rats exhibit methylphenidate-induced cross-sensitization to the behavioral effects of cocaine (adolescent group: Two-way *ANOVA* (drug pretreatment × time): significant drug × time interaction, *F*_(3, 30)_ = 9.1, *p* < .0001; significant differences at 15 min, *p* < .0001; early adult group: Two-way *ANOVA* (drug pretreatment × time): significant drug × time interaction, *F*_(3, 29)_ = 18.1, *p* < .0001; significant differences at 15 - 30 min, *p* < .0001 and at 45 min, *p* < .05). It should be noted that pretreatment with methylphenidate may have altered the baseline locomotor activity of SHR rats, and thus the augmented locomotor response may be a composite of sensitized baseline locomotor activity and sensitized cocaine-induced locomotion. The absence of sensitization in SD and WKY groups is unlikely to be due to increased stereotypy because of the low cocaine dose used to probe for sensitization [21].

These results are not in accord with the conclusion reached by Dafny and colleagues that “the SHR group is the least susceptible to development of behavioral sensitization” [10]. Rather, our results suggest that SHR rats are more vulnerable than other strains to methylphenidate-induced cross-sensitization to the locomotor-activating effects of cocaine when methylphenidate is administered in an environment other than home. While the present results are limited to cross-sensitization of the locomotor-activating effects of methylphenidate and cocaine, our findings suggest that individuals with ADHD that are treated with methylphenidate may have greater vulnerability to abuse illicit drugs than normal individuals, a possibility that has recently been supported [22], but see [23-25].

## Abbreviations

SD: Sprague dawley; SHR: Spontaneously hypertensive rat; WKY: Wistar Kyoto.

## Competing interests

The authors declare that they have no competing interests.

## Authors’ contributions

LY carried out all experimental procedures, performed the data analysis, contributed to the interpretation of the results, and worked with AA to write the manuscript. AA conceived of the study, contributed to the interpretation of the results, and worked with LY to write the manuscript. Both authors read and approved the final manuscript.

## References

[B1] Cabib S Orsini C Le Moal M Piazza PV Abolition and reversal of strain differences in behavioral responses to drugs of abuse after a brief experience Science 2000 9 463 465 10903209 10.1126/science.289.5478.463

[B2] Camp DM Browman KE Robinson TE The effects of methamphetamine and cocaine on motor behavior and extracellular dopamine in the ventral striatum of Lewis versus Fischer 344 rats Brain Res 1994 9 180 193 7704604 10.1016/0006-8993(94)90523-1

[B3] Collins SL Izenwasser S Cocaine differentially alters behavior and neurochemistry in periadolescent versus adult rats Brain Res Dev Brain Res 2002 9 27 34 10.1016/s0165-3806(02)00471-6 12234655

[B4] Giorgi O Piras G Lecca D Corda MG Behavioural effects of acute and repeated cocaine treatments: a comparative study in sensitisation-prone RHA rats and their sensitisation-resistant RLA counterparts Psychopharmacology (Berl) 2005 9 530 538 15772864 10.1007/s00213-005-2177-7

[B5] Haile CN Hiroi N Nestler EJ Kosten TA Differential behavioral responses to cocaine are associated with dynamics of mesolimbic dopamine proteins in Lewis and Fischer 344 rats Synapse 2001 9 179 190 11391778 10.1002/syn.1073

[B6] Laviola G Adriani W Terranova ML Gerra G Psychobiological risk factors for vulnerability to psychostimulants in human adolescents and animal models Neurosci Biobeha Rev 1999 9 993 1010 10.1016/s0149-7634(99)00032-9 10580313

[B7] Laviola G Pascucci T Pieretti S Striatal dopamine sensitization to D-amphetamine in periadolescent but not in adult rats Pharmacol Biochem Behav 2001 9 115 124 11274716 10.1016/s0091-3057(00)00430-5

[B8] Strecker RE Eberle WF Ashby CR Extracellular dopamine and its metabolites in the nucleus accumbens of Fischer and Lewis rats: basal levels and cocaine-induced changes Life Sci 1995 9 PL135 PL141 7530315 10.1016/0024-3205(94)00913-9

[B9] Yang PB Amini B Swann AC Dafny N Strain differences in the behavioral responses of male rats to chronically administered methylphenidate Brain Res 2003 9 139 152 12706230 10.1016/s0006-8993(02)04240-3

[B10] Yang PB Cuellar DO III Swann AC Dafny N Age and genetic strain differences in response to chronic methylphenidate administration Behav Brain Res 2011 9 206 217 21111006 10.1016/j.bbr.2010.11.034

[B11] Yang PB Swann AC Dafny N Acute and chronic methylphenidate dose-response assessment on three adolescent male rat strains Brain Res Bull 2006 9 301 310 17113960 10.1016/j.brainresbull.2006.09.019 PMC2048685

[B12] Vendruscolo LF Izidio GS Takahashi RN Drug reinforcement in a rat model of attention deficit hyperactivity disorder - the spontaneously hypertensive rat (SHR) Curr Drug Abuse Rev 2009 9 183 188 10.2174/1874473710902020177 19630747

[B13] Robinson TE Browman KE Crombag HS Badiani A Modulation of the induction or expression of psychostimulant sensitization by the circumstances surrounding drug administration Neurosci Biobehav Rev 1998 9 347 354 9579324 10.1016/s0149-7634(97)00020-1

[B14] Heidbreder CA Thompson AC Shippenberg TS Role of extracellular dopamine in the initiation and long-term expression of behavioral sensitization to cocaine J Pharmacol Exp Ther 1996 9 490 502 8768696

[B15] Kolta MG Shreve P De Souza V Uretsky NJ Time course of the development of the enhanced behavioral and biochemical responses to amphetamine after pretreatment with amphetamine Neuropharmacology 1985 9 823 829 4058677 10.1016/0028-3908(85)90032-2

[B16] Vanderschuren LJ Schmidt ED De Vries TJ Van Moorsel CA Tilders FJ Schoffelmeer AN A single exposure to amphetamine is sufficient to induce long-term behavioral, neuroendocrine, and neurochemical sensitization in rats J Neurosci 1999 9 9579 9586 10531460 10.1523/JNEUROSCI.19-21-09579.1999 PMC6782918

[B17] Paulson P Robinson T Amphetamine-induced time-dependent sensitization of dopamine neurotransmission in the dorsal and ventral striatum: a microdialysis study in behaving rats Synapse 1995 9 56 65 7709344 10.1002/syn.890190108 PMC1859849

[B18] Vezina P Sensitization of midbrain dopamine neuron reactivity and the self-administration of psychomotor stimulant drugs Neurosci Biobeha Rev 2004 9 827 839 10.1016/j.neubiorev.2003.11.001 15019432

[B19] Pierce RC Kalivas PW A circuitry model of the expression of behavioral sensitization to amphetamine-like psychostimulants Brain Res Brain Res Rev 1997 9 192 216 9403138 10.1016/s0165-0173(97)00021-0

[B20] Robinson T Kolb B Structural plasticity associated with exposure to drugs of abuse Neuropharmacology 2004 9 Suppl 1 33 46 15464124 10.1016/j.neuropharm.2004.06.025

[B21] Kuczenski R Segal DS Aizenstein ML Amphetamine, cocaine and fencamfamine: relationship between locomotor and stereotypy response profiles and caudate and accumbens dopamine dynamics J Neurosci 1991 9 2703 2712 1715389 10.1523/JNEUROSCI.11-09-02703.1991 PMC6575248

[B22] Harvey RC Sen S Deaciuc A Dwoskin LP Kantak KM Methylphenidate treatment in adolescent rats with attention deficit/hyperactivity disorder phenotype: cocaine addiction vulnerability and dopamine transporter function Neuropsychopharmacology 2011 9 837 847 21150910 10.1038/npp.2010.223 PMC3055734

[B23] dela Pena I Hoon J Abuse and dependence liability analysis of methylphenidate in the spontaneously hypertensive rat model of attention-deficit/hyperactivity disorder (ADHD): what have we learned? Arch Pharm Res 2013 9 400 410 23471559 10.1007/s12272-013-0037-2

[B24] dela Pena I Lee JC Lee HL Woo TS Lee HC Sohn AR Cheong JH Differential behavioral responses of the spontaneously hypertensive rat to methylphendiate and methamphetamine: lack of rewarding effect of repeated methylphenidate treatment Neurosci Lett 2012 9 189 193 22414863 10.1016/j.neulet.2012.02.090

[B25] dela Pena I Yoon SY Lee JC dela Pena JB Sohn AR Ryu JH Shin CY Cheong JH Methylphenidate treatment in the spontaneously hypertensive rat: influence on methylphenidate self-administration and reinstatement in comparison with Wistar rats Psychopharmacolgy 2012 9 217 226 10.1007/s00213-011-2564-1 22086360

